# Modelling Sand Fly *Lutzomyia longipalpis* Attraction to Host Odour: Synthetic Sex-Aggregation Pheromone Dominates the Response

**DOI:** 10.3390/microorganisms9030602

**Published:** 2021-03-15

**Authors:** Renata Retkute, Erin Dilger, James G. C. Hamilton, Matt J. Keeling, Orin Courtenay

**Affiliations:** 1 The Zeeman Institute for Systems Biology and Infectious Disease Epidemiology Research (SBIDER), The University of Warwick, Coventry CV4 7AL, UK; Erin.Dilger@warwick.ac.uk (E.D.); M.J.Keeling@warwick.ac.uk (M.J.K.); 2 Epidemiology and Modelling Group, Department of Plant Sciences, University of Cambridge, Cambridge CB2 3EA, UK; 3 School of Life Sciences, The University of Warwick, Coventry CV4 7AL, UK; 4 Division of Biomedical and Life Sciences, Faculty of Health and Medicine, Lancaster University, Lancashire LA1 4YG, UK; j.g.hamilton@lancaster.ac.uk

**Keywords:** *Leishmania*, vector biology, host choice, disease prevention, sex-aggregation pheromone, *Lutzomyia longipalpis*

## Abstract

Zoontic visceral leishmaniasis (ZVL) due to *Leishmania infantum* is a potentially fatal protozoan parasitic disease of humans and dogs. In the Americas, dogs are the reservoir and the sand fly, *Lutzomyia longipalpis,* the principal vector. A synthetic version of the male sand fly produced sex-aggregation pheromone attracts both female and male conspecifics to co-located insecticide, reducing both reservoir infection and vector abundance. However the effect of the synthetic pheromone on the vector’s “choice“ of host (human, animal reservoir, or dead-end host) for blood feeding in the presence of the pheromone is less well understood. In this study, we developed a modelling framework to allow us to predict the relative attractiveness of the synthetic pheromone and potential alterations in host choice. Our analysis indicates that the synthetic pheromone can attract 53% (95% CIs: 39%–86%) of host-seeking female *Lu. longipalpis* and thus it out-competes competing host odours. Importantly, the results suggest that the synthetic pheromone can lure vectors away from humans and dogs, such that when co-located with insecticide, it provides protection against transmission leading to human and canine ZVL.

## 1. Introduction

Understanding the blood-seeking behaviour of arthropod vectors has relevance to vector control against transmission of public and veterinary health diseases [1,2]. The emerging behavioural adaptations of important mosquito vectors to alter their blood-feeding host preferences, locations and feeding time periods, and thus their ability to evade fatal exposure to indoor insecticide interventions [2,3,4], highlight the need for sustained vector surveillance and development of complimentary vector control strategies.

Insect pheromones that mediate mating, aggregation, and invitation behaviours [5], could play a role in maintaining vector contact with insecticides, and consequently in reducing the human biting index. In the agricultural sector, insect pheromones and other semiochemicals have been widely exploited to monitor and reduce pest populations to protect crop yields [5,6,7]. In contrast, pheromones produced by vectors of public or veterinary health importance have not been widely identified or characterised [8] despite their potential to be used to help reduce infection or disease incidence.

One exception is the sand fly *Lutzomyia longipalpis* (Diptera: Psychodidae), which throughout the Americas is the predominant vector of *Leishmania infantum* (Kinetoplastida: Trypanosomatidae), a protozoan parasite that causes human and canine zoonotic visceral leishmaniasis (ZVL) which is usually fatal if not treated [9]. The males of this species produce a sex-aggregation pheromone that mediates attraction of female and male conspecifics to leks for mating [10]. Since leks are usually located on or near to animal hosts, lekking facilitates successful blood feeding by females and thus *Le. infantum* transmission.

*Lu. longipalpis* are catholic in their host choice, feeding on a wide range of non-reservoir (“dead-end”) hosts including household animals, livestock and wildlife species, as well as on domestic dogs which are the sole proven reservoir of *Le. infantum* [11]. *Lu. longipalpis* are not particularly endophagic or endophilic, and are usually trapped in greatest abundance in animal shelters, to which they show a degree of site loyalty as they have a short dispersal range (<100 m) [12,13,14,15]. Dead-end host populations in the near vicinity of human habitation help to maintain the sand fly populations through provision of blood [16], and consequently are likely to influence the epidemiology of ZVL through diversion of infectious vector bites away from humans and the reservoir (zooprophylaxis), or alternatively, by increasing vector densities as the numbers of attractive animals increase (zoopotentiation) [17]. Evidence for these possible outcomes in leishmaniasis epidemiology are currently contradictory or untested [18].

The recent development of a synthetic copy of the male produced *Lu. longipalpis* sex-aggregation pheromone placed in controlled-release dispensers, has provided a unique opportunity to test its attractiveness to conspecific males and blood-seeking females under experimental and field conditions [19,20,21,22,23]. The pheromone has been shown to be attractive at least 30 m from it’s source [23], and can attract many times more females and males to the source than controls [19,20,21,22,23]. Furthermore, co-location in close proximity with pyrethroid insecticides sprayed onto chicken roosting sites or onto household compound perimeter walls in control trials in Brazil, demonstrate that this “lure-and-kill” approach can reduce confirmed canine infection incidence and tissue *Le. infantum* parasite loads by 52–53%, and household vector abundance by 49%–70% [24,25]. A next step is to design and optimise community-wide deployment strategies of this vector control method. However, this requires a good understanding of the potential changes in host choice seeking behaviours in context of competing host odours and variations in demographic and epidemiological conditions.

Mathematical models aid in deciphering important drivers of insect behaviour and predicting changes in epidemiological dynamics when such factors vary. Feeding preferences of sand fly vectors have been modelled explicitely to identify the importance of host defensiveness [26], host irritability [27], host body surface area [28] and host species biomass [29]. However one of the key drivers of vector host-seeking behaviour of *Lu. longipalpis* and possibly other sand fly species is pheromone-mediated lek formation [12,15].

In this study, we report on the development of a mathematical framework to simulate and predict vector blood-seeking behaviour in the context of deploying a synthetic pheromone attractant, parameterised using data from relevant recent field and laboratory studies. To our knowledge this is the first time that the potential of a synthetic pheromone of an arthropod vector has been applied to control a microorganism of public health importance [20,24,25].

## 2. Materials and Methods

**Figure 1:**
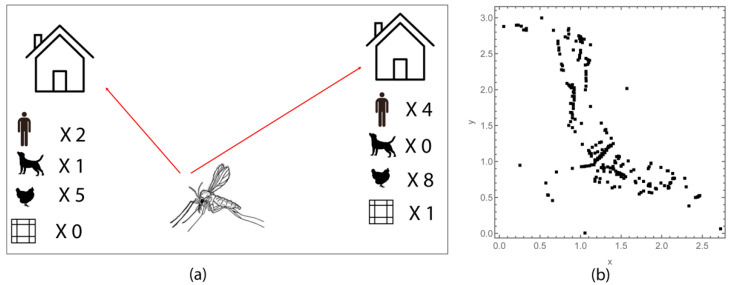
(**a**) Conceptual representation of host choice model: the behaviour of a sand fly depends on the distance and the strength of stimulus received from various sources: hosts (humans, dogs and chickens) and synthetic pheromone traps. (**b**) Location of households [30].

To address the question of the contribution of different attractive elements to the overall attractiveness of a household, we developed a mechanistic spatial model for sand fly host choice in response to a synthetic conspecific sex-aggregation pheromone, whereby the local distribution of sand flies was constructed in terms of attraction profiles. The attraction profile for a household is made up of multiple attractive (and repellant) elements, including hosts (humans, dogs and chickens) and synthetic pheromone traps (Figure 1a). The behaviour of a sand fly responding to a source of attraction depends on the distance required to travel and the strength of competing stimuli received. 

We have defined an attraction profile as:

(1) $$A^{S}\left(d,n,p\right)=exp\left(-p_{1}d\right)p_{2}^{S}\left(1-expa\left(-p_{3}^{S}n\right)\right)$$

where d is the distance, n is either amount of pheromone or a number of hosts; and $p=\left\{p_{1},\;p_{2}^{S},p_{3}^{S}\right\}\;$ are parameters defining the shape of attraction profile for an attractor S={P,H,D,C} , where pheromone (P), human host (H), dog (D) or chicken (C) indicates a type of attractor. We made the following assumptions: (i) attraction decreases with a distance from a source with an exponential decay defined by a parameter $p_{1}$; (ii) there is a saturation effect with increasing amount of pheromone or hosts which is defined by parameter $p_{3}^{S}$; (iii) the height of the attraction profile is equal to $p_{2}^{S}$. 

We have parametrised attraction profiles using data on dispersal of *Lu. longipalpis* [12], host-biting preference [30], and sand fly capture success in synthetic pheromone-baited field traps generated by studies conducted in various locations in Brazil [22,23]. Details of the experiments are given below. 

Dataset A. Capture experiments to assess attraction of *Lu. longipalpis* to the synthetic pheromone [22]. The attraction of individual sand flies to different amounts of pheromone was measured using a series of choice tests. The experiments aimed to test the dose response to the synthetic pheromone relative to chicken-only controls. The quantities of synthetic pheromone tested were 10, 50, 100 or 500 mg and at distances of 5, 10, 15, 20 and 30 m between test and control CDC light traps [22].

Dataset B. Capture–mark–recapture experiments to assess the attraction of *Lu. longipalpis* to the synthetic pheromone [23]. These experiments were conducted using wild-caught sand flies which were marked with fluorescent powders and released at a specific distance from test and control chicken boxes and collected with a modified (no light) CDC trap baited with the synthetic pheromone (10 mg). The synthetic pheromone was placed in the chicken boxes set at distances of 5, 10, 15, 20 and 30 m from the sand fly release point [23] and control traps (without pheromone) were also set at 5, 10, 15, 20 and 30 m from the sand fly release point and 5, 10, 15, 20 and 30 m from the pheromone-baited trap. All chicken boxes (test and control) contained 1 chicken.

Dataset C. Capture experiment to assess host preference [30]. Fieldwork was conducted to investigate the preference of *Lu. longipalpis* for dogs, humans and chickens. CDC light traps were set in three domestic locations: one trap in the bedroom of the house, one in the chicken shed and one trap above a wire mesh cage containing a dog, the relative positions rotated. The relative numbers of sand flies captured per night in each trap were counted [30].

Dataset D. Mark–release–recapture experiment to assess dispersal of *Lu. longipalpis* [12]. Wild-caught sand flies were marked with fluorescent powders and released for recapture at chicken sheds by CDC light trap. The relationship between the percentage of recaptured sand flies at distances (on a natural logarithm scale) was estimated to be proportional to −0.16√d, where d is the distance in meters [12].

A likelihood function for these aggregated data was formulated as a product of binomial distributions for the experimental datasets **A** and **B**, and multinomial distribution for experimental dataset **C**. The probability of success in a single trial was set equal to the probability that a sand fly would be attracted to a source. This probability was calculated by the integration of the convolution between the attraction profile and the dispersal kernel using the MCMC algorithm [31] to estimate the parameters from the aggregated dataset. More details on the likelihood function and fitting procedure are given in Appendix A.

To further understand how the presence of the synthetic pheromone might influence host choice in the heterogeneous landscape, an agent-based mathematical model was designed where a sandfly chooses a household, and a host or pheromone within the household, based on the parametrised attraction profiles. Model simulations were run 1,000 times. The fractions of sand flies attracted to humans, dogs and chickens in each household were calculated running simulations with and without the presence of 10 mg of the synthetic pheromone per household. Variations in the household characteristics were based on the observed geospatial and demographic characteristics of an example rural village (Caldeãro) in Marajó, Pará state, Brazil [30] (Figure 1b). More details on the simulations are given in Appendix B.

## 3. Results

### 3.1. Parametrisation of Attraction Profiles

We fitted attraction profiles to the aggregated dataset containing 309 records from datasets **A**, **B** and **C**. The log-likelihood trace plot for the final 1,000 MCMC iterations and posterior distributions of parameters are shown in supplementary Figure S1.

**Figure 2:**
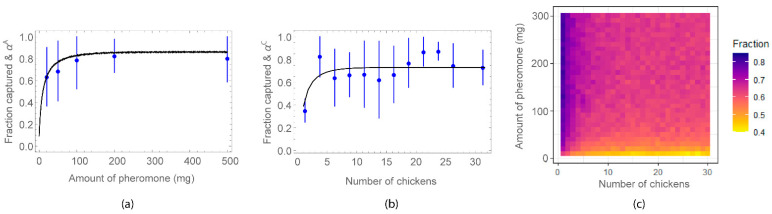
Proportions of *Lu. longipalpis* as a function of: (**a**) amount of pheromone (for the experiment **A**); (**b**) number of chickens (for the experiment **C**); (**c**) amount of pheromone and number of chickens (simulation results). Measured mean and standard deviation are shown in blue and fitted curves in black in (**a**) and (**b**).

The observed fraction of *Lu. longipalpis* captured on chickens, and fitted proportions $\alpha^{A}\;$ (for the experiment **A**) and $\alpha^{C}\;$ (for the experiment **C**) are shown in Figure 2.

The model fits indicate that a greater proportion of female *Lu. longipalpis* would be attracted to a trap baited with the synthetic pheromone relative to a control trap containing a single chicken, where the proportion depends on the amount of pheromone in the trap. Fitted attraction profiles indicate that the number of female *Lu. longipalpis* attracted to the synthetic pheromone can be increased through addition of more pheromone, but the effect saturates at approximately 100 mg (Figure 2a). In the absence of pheromone traps, a similar response can be seen when the number of chickens is increased: the fraction of *Lu. longipalpis* attracted to chicken sheds increases and then saturates at approximately 5 chickens per shed (Figure 2b).

Having a mechanistic model is advantageous to investigate hypothetical and real scenarios which otherwise would require expensive and time consuming field studies. We have simulated capture experiments to measure the effect of increasing the amount of pheromone vs increasing the number of chickens. We have assumed that the virtual experimental setup used one pair of chicken sheds set 30 m apart. The first shed contained a chicken and a different amount of pheromone and the second shed contained different numbers of chickens. The simulated fraction of sand flies attracted to the shed with pheromone is shown in Figure 2c. This fraction was between 0.4 and 0.85. It can be seen that chickens were more attractive only for the minimum amount of pheromone (10–20 mg per trap). In the case of 20 mg, the second shed had to have at least 25 chickens to attract more than 50% of the sand flies. 

### 3.2. Effect of the Synthetic Pheromone on Lu. longipalpis Host Preference

**Figure 3:**
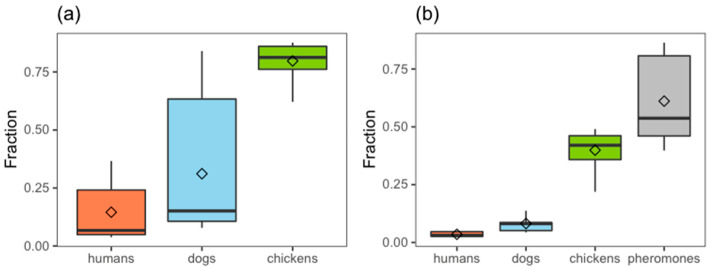
*Lu. longipalpis* estimated preference for hosts and pheromones: (**a**) in the absence of the synthetic pheromone; (**b**) in the presence of the synthetic pheromone.

The estimated effects on host preference in the heterogeneous spatial and demographic landscape is depicted in Figure 3 showing the predicted relative attraction of female *Lu. longipalpis* to the most commonly recorded blood-source hosts: chickens, dogs and humans, in the absence (3a) and presence (3b) of the synthetic pheromone. The variability in the fraction of *Lu. longipalpis* attracted to each source arises from the observed heterogeneity in host demography, i.e., the numbers of humans, dogs and chickens, recorded per household [29,30].

Across all households, in the absence of the synthetic pheromone, a median 6.7% (95% CIs: 3.8%–36.6%) of sand flies are preferentially attracted to humans, 15.1% (95% CIs: 7.8%–84.0%) to dogs, and 81.2% (95% CIs: 54.4%–87.6%) to chickens. In the presence of 10 mg of the synthetic pheromone, the expected median proportion of sand flies attracted to each of the three hosts decreased by approximately half: 3.1% (95% CIs: 2.2%–4.9%) (humans), 8.1% (95% CIs: 4.3%–22.2%) (dogs), and 42.1% (95% CIs: 14.8%–49.1%) (chickens), whereas the attraction to the synthetic pheromone accounted for 53.7% (95% CIs: 39.7%–86.4%) of total *Lu. longipalpis* (Figure 3b).

## 4. Discussion

We modelled the localised influence of the synthetic pheromone in treated households based on empirical data showing that it can attract conspecifics from at least 30 m away [23], and that the attraction strength is non-linearly related to the pheromone quantity (dose dependent) [22]. When constructing the attraction profiles, we assumed that attraction decreases exponentially with distance from a source, and a saturation effect is reached as the amount of the synthetic pheromone or numbers of hosts (kairomone quantity) increases [15,22,23]. We introduce the term “attraction profile” to incorporate the synthetic pheromone as a potential “choice” for sand flies, in contrast to malaria models where the term “attraction rate” refers to the blood-meal choice dependence on the propensity of hosts to emit kairomone attractants, and on host accessibility [17]. Here we present an extension to the vector-host interaction model framework to inform interventions that include vector attractants, in this case a synthetic vector pheromone which can be co-located with insecticide as a “lure-and-kill” method against a public health disease. Our proposed mathematical framework explicitly models the quantitative interactions between the synthetic pheromone and key types of host (dead-end, reservoir and humans) within a realistic heterogeneous spatial and demographic dimension. Other influencing factors such as host accessibility [32,33] could easily be encorporated into the model framework. 

The current simulations indicate that in the absence of the synthetic pheromone, chickens would be expected to be the preferred host (81.2%), with dogs being second choice (15.1%) and humans the least attractive (6.7%). This is expected as many studies demonstrate that *Lu. longipalpis* is trapped at greatest densities in animal shelters compared to inside houses [13,33]. In households fitted with the synthetic pheromone, model simulations predict that the synthetic pheromone attracts female *Lu. longipapis* away from the three potential alternative hosts, and in approximately similar proportions (46.3–53.6%), indicating substantial reductions in the absolute biting rates on humans and the canine reservoir. There is no evidence from community studies that the synthetic pheromone attracts larger numbers of *Lu. longipalpis* to households which could lead to zoopotentiation [24]. On the contrary, when co-located with insecticide, the pheromone can reduce vector numbers even in neighbouring houses which do not receive the lure-and-kill intervention [25]. Locating the synthetic pheromone and insecticide within household compounds contrasts to that of experimental human odour lures to attract mosquitoes, which are longer range attractants, and therefore best placed further away from human residencies and other mosquito aggregation sites in order to reduce the risk of zoopotentiation [34].

The model outcomes clearly demonstrate that the preference of *Lu. longipalpis* is skewed towards the synthetic pheromone within the context of competing host odours and the naturally-released male pheromone, but it appears not to alter the relative preference for the three types of hosts. A similar finding is observed from data independent from those used to parameterise the current model, collected elsewhere in Brazil where CDC traps were placed in houses, chicken roosting sites and above tethered dogs, in control and treated households [24]. In that study, the lure-and-kill approach reduced transmission in the canine reservoir by approximately 50% but did not increase household vector abundance [24].

The feasibility of a lure-and-kill strategy for community-wide deployment will depend on economic evaluation as an important consideration for public policy decision making. A key motivation of this study was the need to provide a mathematical framework to predict the likely outcomes on host choice underpinning field trial results, but it also serves as a framework to help optimise alternative interventions with spatial modes of action. The next steps will be to incorporate the predictive mathematical model of vector host-seeking behavior with spatially explicit VL transmission models to evaluate changes in human and canine spatial infection incidence under variable pheromone implementation scenarios and demographic conditions.

In conclusion, our analysis indicates that when the synthetic pheromone (10 mg) is co-located with insecticide, the lure-and-kill approach could dilute mean vector biting rates on humans and the canine reservoir by approximately half.

## Data Availability

The data supporting the conclusions of this article are included within the article. Data are freely available from DOI 10.5281/zenodo.4602849 on request to be used solely within the context of this study, following the ethical agreements and permissions granted from the relevant authorities for the conduct of this study.

## Supplementary Materials

The following are available online a https://www.mdpi.com/2076-2607/9/3/602/s1, Figure S1: MCMC chain and posterior distribution of parameters. Table S1: Household characteristics.

## Appendix A

The dispersal was evaluated through the mark–release–recapture method [12]. The relationship between the percentage of recaptured sand flies (Iog scale) has been estimated to be proportional to $-0.16\sqrt{d}\;$, where *d* is the distance in meters. Consequently, we have set the dispersal kernel function as:

$$K\left(d\right)=K_{0}exp\left(-0.16\sqrt{d}\right)$$

where

$K_{0}={\left(\int_{0}^{\infty }\exp\left(-0.16\sqrt{x}\right)dx\right)}^{-1}\;$.

A proportion of sand flies attracted to a household *h* with $n_{S}\left(t\right)$ number of attractors at time *t* can be calculated by summing the integrals:

$$f_{h}\left(t\right)\;=\;\frac{\sum_{S\in \left\{P,H,D,C\right\}}\int_{0}^{\infty }K\left(x\right)A^{S}\left(\left|x^{h}-x\right|,n_{S}\left(t\right),p^{S}\right)dx}{F\left(t\right)}\;,$$

where *S* is source, {*P*, *H*, *D*, *C*} stands for pheromone lure, human, dog and chicken; *K*(*x*) is the dispersal kernel, attraction profile is given by Equation (1), and *x^h^* is coordinate of the household *h*.

Normalisation is obtained by summing over all households, i.e.,

$$F\left(t\right)\;=\;\sum_{h}\sum_{S\in \left\{P,H,D,C\right\}}\int_{0}^{\infty }K\left(x\right)A^{S}\left(\left|x^{h}-x\right|,n_{S}\left(t\right),p^{S}\right)dx$$

We can also calculate a fraction of sand flies attracted to a particular host in a household *h*, for example for dogs:

$$f_{h}^{D}\left(t\right)\;=\;\frac{\int_{0}^{\infty }K\left(x\right)A^{D}\left(\left|x^{h}-x\right|,n_{D}\left(t\right),p^{S}\right)}{F\left(t\right)}\;.$$

There are nine parameters defining attraction profiles:$\left\{p_{1},p_{2}^{P},p_{3}^{P},p_{2}^{H},p_{3}^{H},p_{2}^{D},p_{3}^{D},p_{2}^{C},p_{3}^{C}\right\}\;$, where the upper indices are for pheromone (P), human hosts (H), dogs (D) or chickens (C), and the lower indices correspond to decays with distance (1), relative height of the profile (2) and saturation with amount (3). We set $p_{2}^{P}=1\;$, i.e., the height of the attraction profile for a maximum amount of pheromone to be equal to one.

Below we describe how the likelihood function for each of the experiment has been formulated.

In the experiment **A**, there were two chicken sheds positioned at $x^{test}$ and $x^{control}$ sites. In each shed, there was a synthetic pheromone trap equipped with $\varphi_{i}$ (*i* = {*contr*, *test*}) amount of the synthetic pheromone, where $\varphi_{i}\;=\;0$ corresponds to the control with no synthetic pheromone present. We set up a virtual experiment within a rectangle around a control and test trap and assumed that sand fly can enter at a random point along the perimeter. The proportion of sand flies attracted to the test trap is calculated as:

$$\alpha_{j}^{A}\;=\;\frac{\beta \left(d_{test},\varphi_{test},x^{test}\right)}{\beta \left(d_{test},\varphi_{test},x^{test}\right)\;+\;\beta \left(d_{control},\varphi_{control},x^{control}\right)}\;,$$

$$\beta \left(d,\varphi ,y\right)\;=\;\int_{0}^{d}K\left(x\right)A\left(\left|y\;-\;x\right|,\varphi ,n_{C},p\right)dx/d.$$

where $d_{test}$ is the distance between the test trap and entry point; $d_{control}$ is the distance between a control trap and entry point. As a chicken was placed in each shed to provide a source of host odour, we assume that:

$$A\left(\left|y\;-\;x\right|,\varphi ,n_{C},p\right)\;=\;A^{P}\left(\left|y\;-\;x\right|,\varphi ,p\right)\;+\;A^{C}\left(\left|y\;-\;x\right|,1,p\right),\;$$

i.e., the attraction profile is a sum of attraction profiles for the synthetic pheromone and chickens with $n_{C}\;=\;1$. We run *r* simulations with randomly positioned entry point along perimeter of the rectangle and set $\alpha^{A}\;=\;\frac{1}{r}\sum_{j=1}^{r}\alpha_{j}^{A}\;$.

In experiment **B**, sand fly was captured, colour coded, released and recaptured. We assumed that released sand flies which were not recaptured in the test trap have dispersed elsewhere. Therefore, the proportion of sand flies attracted to the test trap was calculated as

$$\alpha^{B}\;=\;\frac{\int_{R^{2}}K\left(y\right)A\left(\left|x\;-\;y\right|,\varphi ,n_{C},p\right)dy}{\int_{R^{2}}K\left(y\right)A\left(\left|x\;-\;y\right|,\varphi ,n_{C},p\right)dy\;+\;\int_{R^{2}}K\left(y\right)dy}.$$

In experiment **C**, the proportions of sand flies attracted to humans, dogs and chickens are calculated as:

$$\alpha_{s,i}^{C}\;=\;\frac{\left(1/d_{s}\right)\int_{0}^{d_{s}}K\left(x\right)A\left(\left|x^{s}\;-\;x\right|,n_{s},p\right)dx}{\sum_{s\in S}\int_{0}^{d_{s}}K\left(x\right)A\left(\left|x^{s}\;-\;x\right|,n_{s},p\right)dx},$$

where *S* = {*H*;*D*;*C*}, *d_S_* is the distance between a trap S and entry point, *n_S_* is a number of individuals of type *S*. We run *r* simulations with randomly positioned entry point and

$$\alpha^{C}\;=\;\frac{1}{r}\sum_{j=1}^{r}\alpha_{s,i}^{C}\;.$$

Finally, we assume that the number of sand flies caught in a trap follows a binomial distribution with success probability equal to $\alpha^{A}\;$ for the experiment **A**, $\alpha^{B}\;$ for the experiment **B**, and multinomial distribution with probabilities $\alpha^{C}\;$ for the experiment **C**. Then the likelihood function is given by:

$$\begin{array}{c} L\left(p\right)\;=\;\prod_{i=1}^{n_{A}}Binom\left(n_{i}^{test},n_{i}^{test}\;+\;n_{i}^{control},\alpha^{A}\right)\;\times \;\overset{n_{B}}{\underset{i=1}{\prod }}Binom\left(n_{i}^{recapture},n_{i}^{release},\alpha^{A}\right)\\ \times \;\overset{n_{C}}{\underset{i=1}{\prod }}Multinom\left(n_{i}^{C},n_{i}^{D},n_{i}^{H},n_{i}^{C}\;+\;n_{i}^{D}\;+\;n_{i}^{H},\alpha^{C}\right). \end{array}\;$$

We used the MCMC algorithm to estimate the parameters from 309 available datasets. We set a number of entry points to *r* = *10^5*. We chose uniform priors U[0; 1] for all parameters. Parameters were updated using an adaptive random walk Metropolis algorithm with proposal distribution given at iteration *k* [31]:

$$Q_{k}\left(x,.\right)=\{\begin{array}{c} N\left(x,0.1^{2}I_{m}/m\right)\;if\;\;k\;\leq \;2m,\\ \left(1\;-\;\xi \right)N\left(x,\;2.38^{2}\Sigma_{m}/m\right)\;+\;\xi N\left(x,0.1^{2}I_{m}/m\right),\;\;\;\;otherwise. \end{array}\;$$

where *m* = 8 is a number of parameters to estimate, $\Sigma_{m}$ is an empirical estimate of the covariance matrix at iteration k, and ξ = 0.05 .

## Appendix B

The spatial and demographic data for a rural setting came from the fieldwork and survey data from Calderao village, Marajó [30]. The data consisted of GPS coordinates of households, and the number of humans, dogs and chickens resident in the household. Households with missing data for human numbers were assigned a median of five closest neighbours, and number of chickens were sampled from the Marajó dataset. The latitude and longitude coordinates of households were converted to Universal Transverse Mercator zone 22 projection and translated so that the left bottom corner of a local coordinate system is (0,0). There were 235 households in the village. Locations of the households are shown in Figure 1b. Table S1 gives statistics on demographic data.

To account for a local impact of synthetic pheromone traps, we apply following steps:

1. We calculate a number of sand flies in each household without pheromone traps $V_{h}$.

2. For each household with a pheromone lure, we redistributed sand flies to pheromone lure and hosts according to a ratio between attraction profiles:

$$f_{s}\;=\;\frac{\int_{0}^{\infty }K\left(x\right)A^{s}\left(\left|x^{s}\;-\;x\right|,n_{s},p^{s}\right)dx}{\sum_{S\in \left\{P,H,D,C\right\}}\int_{0}^{\infty }K\left(x\right)A^{S}\left(\left|x^{S}\;-\;x\right|,n_{S},p^{S}\right)dx}.\;$$

3. Number of sand flies attracted to a host after introduction of the synthetic pheromone is equal:

$$V_{h}^{S}\;=\;f_{S}V_{h}\;.$$

We set total number of female sand flies to $V_{h}\;=\;1000\;$. We calculate sand flies attracted to hosts and pheromones in each household and aggregate these proportions in order to draw a distribution. The boxplots for estimated sand fly preferences for hosts and pheromones in the absence and presence of pheromone traps are shown in Figure 3.
