# Supplementary material for: Modelling Sand Fly Lutzomyia longipalpis Attraction to Host Odour: Synthetic Sex-Aggregation Pheromone Dominates the Response
Source: Microorganisms. 2021 Mar 15;9(3):602. doi: 10.3390/microorganisms9030602 (PMC7999287; doi:10.3390/microorganisms9030602)
Supplement: Supplementary file 1 [file microorganisms-09-00602-s001.pdf]

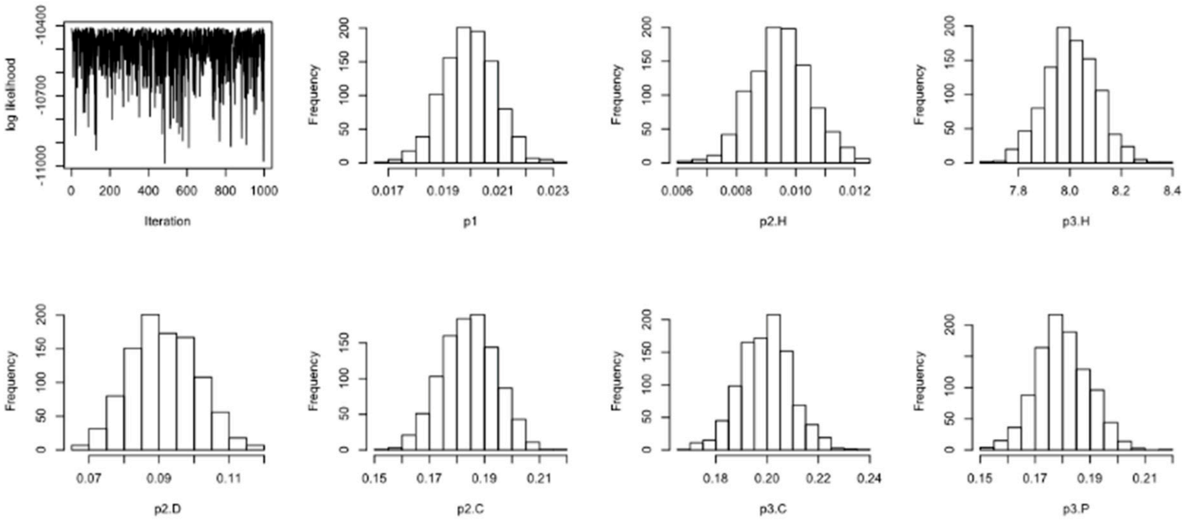

Figure 1. MCMC chain and posterior distribution of parameters.

Table 1. Household characteristics

Characteristics	Value
Number of households	235
Average number of humans per household	4.67
Average number of dogs per household	1.09
Average number of chickens per household	6.8
